# Genealogy-based trait association with LOCATER boosts power at loci with allelic heterogeneity

**DOI:** 10.1101/gr.280372.124

**Published:** 2026-05

**Authors:** Xinxin Wang, Ryan Christ, Erica Young, Chul Joo Kang, Indraniel Das, Edward A. Belter, Markku Laakso, Louis J.M. Aslett, David Steinsaltz, Nathan O. Stitziel, Ira M. Hall

**Affiliations:** 1 Department of Genetics, Yale University, New Haven, Connecticut 06520, USA;; 2 Center for Genomic Health, Yale University, New Haven, Connecticut 06520, USA;; 3 Roy and Diana Vagelos Division of Biology and Biomedical Sciences, Washington University School of Medicine, Saint Louis, Missouri 63110, USA;; 4 Center for Cardiovascular Research, Division of Cardiology, Department of Medicine, Washington University School of Medicine, Saint Louis, Missouri 63110, USA;; 5 McDonnell Genome Institute, Washington University School of Medicine, Saint Louis, Missouri 63108, USA;; 6 Institute of Clinical Medicine, Internal Medicine, University of Eastern Finland, Kuopio FI-70211, Finland;; 7 Department of Mathematical Sciences, Durham University, Durham DH1 3LE, United Kingdom;; 8 Department of Statistics, University of Oxford, Oxford OX1 4BH, United Kingdom;; 9 Department of Genetics, Washington University School of Medicine, Saint Louis, Missouri 63110, USA

## Abstract

A key methodological challenge for genome-wide association studies is how to leverage haplotype diversity and allelic heterogeneity to improve trait association power, especially in noncoding regions where it is difficult to predict variant impacts and define functional units for variant aggregation. Genealogy-based association methods have the potential to bridge this gap by testing combinations of common and rare haplotypes based purely on their ancestral relationships. In parallel work, we have developed an efficient local ancestry inference engine and a novel statistical method (LOCATER) for combining signals present on different branches of a locus-specific haplotype tree. Here, we develop a genome-wide LOCATER analysis pipeline and apply it to a genome sequencing study of 6795 Finnish individuals with 101 cardiometabolic traits and 18.9 million autosomal variants. We identify 351 significant trait associations at 47 distinct genomic loci and find that LOCATER boosts the single marker test (SMT) association signal at five loci by combining independent signals from distinct alleles. LOCATER successfully recovers known quantitative trait loci not found by SMT, including *LIPG*, recovers known allelic heterogeneity at the *APOE/C1/C4/C2* gene cluster, and suggests one novel association. We find that confounders have a more pronounced effect on genealogy-based methods than SMT, and we propose a new randomization approach and a general method for genomic control to eliminate their effects. This study demonstrates that genealogy-based methods such as LOCATER excel when multiple causal variants are present and suggests that their application to larger and more diverse cohorts will be fruitful.

Genome-wide association studies have been extremely successful at identifying variants and genes associated with common human diseases and other complex traits. The vast majority of studies have used one or both of two statistical methods for trait association: common variant association using single marker tests (SMTs) and rare variant association using gene-based aggregation tests (Lee et al. 2014; Povysil et al. 2019). However, neither method is well suited for identifying rare variant associations in noncoding regions where most causal variants are known to reside (Maurano et al. 2012) or for testing the combined association of multiple independent common and/or rare variant signals (potentially with opposing effects) in cases of allelic heterogeneity (i.e., when multiple causal variants are present at a locus). Region-based methods have been adapted to noncoding regions using sliding window approaches, with some success (Li et al. 2019), but this approach is limited by two major challenges. First, it is difficult to decide which intervals and sets of variants to test in noncoding regions where knowledge of variant function is limited. Second, including nonfunctional variants in these tests can greatly reduce power.

In theory, genealogy-based methods that seek to associate local ancestral clades with traits have the potential to overcome these limitations through their ability to combine independent and potentially opposing signals present in different regions of the local ancestral tree, without the need to define functional regions or variant sets. Despite notable early progress (Zöllner and Pritchard 2005; Minichiello and Durbin 2006), these methods have proven difficult to implement in practice owing to the computational challenges of genome-wide population-scale haplotype inference and the statistical challenges of tree-based association testing. Recent advances in haplotype inference have eased the computational burden of building local genealogies (Rasmussen et al. 2014; Kelleher et al. 2019; Speidel et al. 2019; Zhang et al. 2023; Aslett and Christ 2024), making genome-wide trait association studies feasible (albeit still computationally expensive). Building on this, several new methods have recently been developed to test genome-wide local genealogies for trait association.

The first, ARG-Needle (Zhang et al. 2023), builds local genealogies using a scalable ancestral recombination graph–based algorithm, samples a set of inferred clades that may harbor an unobserved variant, and adds the genotypes corresponding to those clades to the list of genotypes tested in genome-wide SMT. Although ARG-Needle is an extremely powerful method for reference free imputation, this “inferred variant” SMT approach is not designed to combine independent genetic signals at loci with allelic heterogeneity.

A second method (Link et al. 2023) uses the Li and Stephens (LS) model HMM implemented in Relate (Speidel et al. 2019) to generate local expected genetic relatedness matrices (eGRMs), which are then tested for association with the phenotype. Link et al. (2023) showed via simulations that this approach can boost power when multiple independent causal variants are present at a locus, and their analysis of two chromosomes in a Native Hawaiian cohort of 5384 individuals found evidence for a robust (albeit not genome-wide significant) BMI association signal at the *CREBRF* locus. A more recent method (Gunnarsson et al. 2024; Zhu et al. 2024) uses a highly scalable local ancestry inference engine and a statistical testing approach similar to that of Link et al. (2023) and has been applied to gene-based trait association in the UK Biobank. However, because both of the above methods are based purely on a quadratic form test statistic, association signals driven by a small number of clades can be easily diluted by other structures in the local relatedness matrix.

Our method, LOCATER (Christ et al. 2026), employs a novel three-step procedure to test for the association of local genealogical relationships with traits, including a traditional SMT, the newly developed stable distillation (SD) association test (Christ et al. 2022), and a quadratic form (QForm) association test similar to the method used by Link et al. (2023). SD is a specialized association test that is much more powerful than quadratic form testing at assessing the combined effects of many small clades marked by ultrarare variants. When used in conjunction with an optimized implementation of the LS model we developed to infer local ancestry, kalis (Aslett and Christ 2024), our simulation studies have shown that LOCATER outperforms SMT in cases of allelic heterogeneity, that this advantage is more pronounced as the number of causal variants increases and their allele frequency decreases, and that the SD subtest is the primary driver of power gains (Christ et al. 2026).

Recent evidence suggests that allelic heterogeneity is widespread in humans. For example, a recent massive parallel reporter assay (MPRA) study estimated that 10%∼20% of expression quantitative trait loci (eQTLs) have multiple causal variants in humans (Abell et al. 2022), and a previous study showed that by inference, the proportion of all loci with allelic heterogeneity is 4% to 23% in eQTLs, 35% in GWASs of high-density lipoprotein (HDL), and 23% in GWASs of schizophrenia (Hormozdiari et al. 2017). These and related observations (The GTEx Consortium 2020) suggest that methods such as LOCATER that leverage allelic heterogeneity to improve power have the potential to discover novel trait associated alleles and genes not found by other methods.

Here, we use LOCATER to screen for trait-associated loci in the METSIM cohort. METSIM is a population-sampled cohort of Finnish men with whole-genome sequencing (WGS) data and a large number of cardiometabolic traits. Prior genome-wide association studies in METSIM have mapped many loci associated with cardiometabolic traits and disease, including studies based on array-based genotype data (Davis et al. 2017; Karjalainen et al. 2024), exome sequencing data (Locke et al. 2019), and WGS data (Chen et al. 2021; Ganel et al. 2021). Because of a recent population bottleneck and subsequent expansion, genetic diversity is somewhat reduced in Finland and there is a larger fraction of deleterious variants at intermediate allele frequencies, facilitating trait mapping at relatively modest sample sizes (Manolio et al. 2009; Locke et al. 2019). These features, coupled with extensive prior knowledge of Finnish genetics, make this an ideal cohort to test new trait mapping methods such as LOCATER.

## Results

The LOCATER method has been described in detail and tested on simulated data in separate work (Christ et al. 2026). Briefly, LOCATER involves a three-step process, the first of which is to run local ancestry inference at each genetic marker. LOCATER uses an optimized version of the LS model and represents local ancestry as matrices (Speidel et al. 2019). The second step is to identify small clades, which we refer to as “sprigs.” Sprigs are independent predictors that were derived from the smallest possible inferred clades. LOCATER tests discretely called clade genotypes in X(ℓ) and clades encoded in the local relatedness matrix in Ω(ℓ). As in the work of Christ et al. (2026), we use X(ℓ) to only encode very small clades (sprigs): Each typically has at most 10 haplotypes under it. LOCATER includes a sprig-calling algorithm that clusters the genomic distances and calls sprigs with a greedy clique–finding procedure. Then, at each genetic marker, LOCATER performs three association tests sequentially: (1) a standard SMT to measure the contribution of that specific marker, (2) Stable Distillation (SD) to measure the contribution of the inferred small clades based on inferred local ancestry, and (3) QForm to measure the contribution of remaining ancestral relationships (Christ et al. 2026). Steps 2 and 3 use a residualized phenotype vector from the prior step. This, combined with the independence guarantees of SD (Christ et al. 2022), ensures that the resulting three *P*-values from SMT, SD, and QForm are mutually independent under the null hypothesis. We then combine the three *P*-values using an adapted version of Fisher's method that we call Maximizing over Subsets of Summed Exponentials (MSSE) (Christ et al. 2026). Multiple optimizations for computing efficiency were implemented throughout the LOCATER pipeline, with more details in the work of Christ et al. (2026) and in the Methods.

Applying LOCATER in this study required overcoming two key methodological challenges that were not addressed in our prior simulation-based work (Christ et al. 2026): tuning our ancestry inference engine for use with real-world WGS data, specifically from the METSIM cohort in Finland, and fully accounting for the effects of cryptic confounders in haplotype screening. Although the specific details of these analysis steps will need to be worked out for any new association study that uses LOCATER or similar methods on a new data set, the approaches we describe below may provide a general solution that helps guide future implementation of these methods.

### Parameter tuning for local ancestry inference

The LOCATER pipeline used for our prior and current work uses the Speidel version (Speidel et al. 2019) of the LS model implemented in kalis (Aslett and Christ 2024), which includes recent developments to improve efficiency for genome-wide testing (Christ et al. 2026). As described by Aslett and Christ (2024), the LS model in kalis has two parameters: the effective population size parameter (N_e_), and the mutation probability (μ). Because N_e_ acts by rescaling the provided recombination map in the LS model (Equation 3) (Aslett and Christ 2024), we will refer to −log_10_N_e_ as the recombination penalty parameter. Analogously, we will refer to −log_10_µ as the mutation penalty parameter.

To best represent local genealogy structure and propagate association signals, tuning these parameters has been a focus of recent work on the LS model (Speidel et al. 2019; Jin and Terhorst 2023). Rather than using the data likelihood and expectation-maximization (EM) (Baum et al. 1970) to select these two parameters for our METSIM analysis, Relate (Speidel et al. 2019) proposes using a more relevant objective aimed at maximizing the performance of the LS model for their specific purpose: capturing local variants in their inferred ancestral trees. Here, we propose an objective aimed at optimizing the discovery power of LOCATER and other methods aiming to leverage allelic heterogeneity. At a given locus, LOCATER works on matrices representing inferred local genealogy derived from the modified LS model, and LOCATER boosts the power of SMT by applying SD and QForm to the local genealogy representations (Christ et al. 2026). Thus, we selected HMM parameters that optimize the propagation of nearby signals using the following objective function and sampling scheme.

We randomly sampled many genomic regions from the data set and assigned a causal variant and a target variant that are 0.05 cM away (for details, see Methods). We then simulated a phenotype vector with a strong effect driven by the causal variant and ran LOCATER at the target variant to measure how well it could capture the signal driven by the nearby causal variant. We calculated the relative signal as our metric, defined as

$$\frac{-\log(P_{SD}^{l}P_{Q}^{l})}{-\log(P_{SMT}^{m})},$$

where *l* is the index of the target variant and *m* is the index of the causal variant. *P*_*SMT*_ refers to *P*-values returned by SMT, and *P*_*SD*_ and *P*_*Q*_ refer to *P*-values returned by the SD and QForm subtests in LOCATER, respectively. This objective focused on association signal propagation could be used to train parameters for any association method targeting loci with allelic heterogeneity.

The trimmed mean surface for this relative signal showed that multiple parameters have comparable efficiency and formed a plateau (see Methods) (Supplemental Fig. S1; for HMM parameters evaluated, see Supplemental Table S3). Our result aligns with a similar finding in the work of Speidel et al. (2019) that high mutation penalties combined with low recombination penalties are not well suited for haplotype inference. After ruling out parameters that require a much longer time to run, we randomly picked one parameter (recombination penalty of six and a mutation penalty of eight) on the plateau of the surface, averaging across allele frequency bins. We also generated surfaces for different allele frequency bins and confirmed that the shape of the surfaces remains consistent across allele frequency bins for both the QForm and SD association methods.

### Methodological improvements to the LOCATER pipeline to account for cryptic confounders

In our initial trait association experiments, we observed deviations in the empirical *P*-value distributions from LOCATER, with cases of both mild inflation and mild deflation across the 101 traits analyzed in this study, despite using standard procedures to correct for population structure using principal component analysis (PCA). In contrast, we did not observe inflation when running SMT on the exact same data, nor did we observe inflation when running LOCATER on simulated phenotype data. This suggests that genealogy-based trait association methods are especially sensitive to the effects of cryptic confounders. We believe that this is because there is a much greater degree of correlation between nearby genetic markers for tree-based tests than for SMT and that this causes a much larger fraction of markers to be affected by confounders. Notably, this implies that these confounders are also affecting the SMT results, just in a less readily detectable way.

To more precisely calibrate LOCATER results while maximizing power, we took inspiration from the phenotype rank-matching procedure used in LOCATER. By default, LOCATER normalizes phenotypes by mapping ranks to simulated Gaussian random variables rather than to fixed quantiles (Christ et al. 2026). This approach avoids the subtle dependence induced when mapping to fixed quantiles. Under the assumption that the original residuals are exchangeable, matching to simulated Gaussian random variables indeed yields independent Gaussian phenotypes, which is assumed by the SD procedure underlying LOCATER.

Building on this procedure, we repeated the rank-matching process for the same phenotype several times, each version of the phenotype vector is a subtly different perturbation of the original phenotype. We found that when we ran LOCATER across these different perturbations, the amount of inflation observed via Q–Q plots differed moderately across perturbations. We believe this variation reflects the fact that, by chance, different perturbations can have stronger or weaker correlations with confounding factors such as population structure. Thus, the rank-matching procedure allows us to modulate the correlation between the phenotype and confounding variables such that we can identify a rank-matched version that is the least correlated with confounders. This approach is similar to that of surrogate variable analysis (Leek and Storey 2007), which is commonly used in the analysis of gene expression data. We therefore rank matched each phenotype 100 times and chose the version that showed the least alignment with confounders for our modified genomic control (GC; see Methods). For each version of each phenotype, we conducted an association test for about 30,000 evenly spaced variants and selected the version for which the *P*-value distribution is the closest to a uniform distribution for both SD and QForm (see Methods). We found that SMT *P*-values of all versions are consistently well calibrated, and SD and QForm *P*-values from different versions will have substantially different deviations from the theoretical null (Supplemental Figs. S2–S4, columns “Rank matched chosen”, “Rank matched 2”, and “Rank matched 3”). We also found that the rank-matched phenotype chosen for the association study indeed has the least deviation and eliminated the inflated body of the QForm *P*-value distribution. Our study is the first to report genome-wide Q–Q plots for a genealogy-based association method and demonstrate calibration of that method after rigorously adjusting for unobserved confounding factors.

We then sought to apply a more general GC to the *P*-values. In short, we fit a line to the Q–Q plot of LOCATER −log_10_p of the selected rank-matched phenotype. We applied modified GC to *P*-values using the slope and intercept of the fitted line (see Methods). This is similar to traditional GC (Devlin and Roeder 1999), which only fits a slope to the Q–Q plot. By incorporating a nonzero intercept, we can achieve a much more accurate fit to the tail of our null distribution of −log_10_p. To gain a better intuition for the role of this intercept, it is helpful to think in terms of a simple application like single-marker testing in which each *P*-value corresponds to a *Z*-score. In this setting, fitting a slope via classic GC to the Q–Q plot is equivalent to fitting a scale parameter to the distribution of null *Z*-scores while keeping the location of that null distribution fixed at zero. Introducing a nonzero intercept allows one to fit a null distribution to the *Z*-scores in which the location may also be adjusted. A similar procedure is described in chapter 6 of the work of Efron (2010) and is standard in large-scale testing problems in which the null assumptions are not satisfied (e.g., when there is confounding). In Supplemental Figures S2–S4, we applied this modified GC procedure to *P*-value distributions from LOCATER-specific tests, and all distributions align with the expected distribution much better (Supplemental Figs. S2–S4, rows “SD modified GC” and “QForm modified GC”).

### GWAS of 101 traits

**Figure 1.**
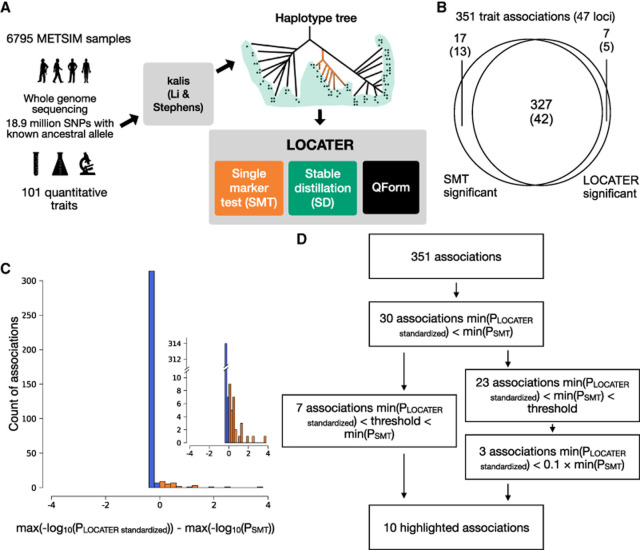
Schematic of our genealogy-based screening procedure LOCATER and summary of screening results. (*A*) Diagram of the experimental design and association testing method. kalis is an implementation of the Li and Stephens (LS) model for local ancestry inference. (QForm) Quadratic form testing. (*B*) Venn diagram showing the number of associations and number of loci (in parentheses) with significant SMT and/or significant LOCATER results. Note that a given locus may have distinct associations represented in different parts of the Venn diagram. (*C*) Distribution of max(−log_10_(P_LOCATER standardized_)) − max(−log_10_(P_SMT_)) for all 351 associations. All associations that are genome-wide significant by either SMT or LOCATER are included. (*D*) Overview of significant associations and highlighted associations. The genome-wide significance threshold is 7.17 × 10^−9^ for SMT and standardized LOCATER.

We then performed an association analysis of 6795 METSIM individuals with 101 quantitative metabolic phenotypes (Fig. 1A; for highlighted associations, see Table 1) using LOCATER. We also analyzed these traits with SMT as our benchmark. All traits were residualized based on trait-specific covariates beforehand, exactly as described in our prior exome based study of this same cohort (Locke et al. 2019). After filtering out indels and variants lacking ancestral allele annotation (see Methods), we tested 18,949,137 autosomal variants. We used the top 10 Finnish-specific principal components (PCs) as background covariates for the association test. As mentioned above, during the prescreening stage, we selected the phenotype vector perturbation that yielded the most calibrated Q–Q plot (see Methods). Postscreening, we applied modified GC to the resulting *P*-values based on the slope and intercept (as described above; see Methods) in the Q–Q plot to ensure that both SD and QForm subtests in LOCATER were well calibrated.

**Table 1.** Summary of highlighted associations

Variant ID	Mapped gene	Trait	LOCATER *P* (modified GCed)	LOCATER lead marker MAF	Variant ID of SMT lead marker	SMT *P*	SMT lead marker MAF	Known hit in GWAS catalog	SMT lead marker LD with GWAS catalog marker
Chr7-73,440,219-C-T	*FZD9, BAZ1B*	MUFA	1.57 × 10^−9^	0.096	Chr7-73,440,219-C-T^a^	2.52 × 10^−8^	0.096	Yes	0.701
Chr7-73,482,065-A-C	*BAZ1B*	Triglycerides in VLDL	1.64 × 10^−11^	0.12	Chr7-73,467,477-C-T	1.11 × 10^−9^	0.12	Yes	0.852
Chr7-73,643,687-A-G	*VPS37D, MLXIPL*	Concentration of large VLDL particles	2.82 × 10^−9^	0.12	Chr7-73,641,131-A-C	3.40 × 10^−8^	0.12	Yes, related trait	0.933
Chr11-61,843,278-G-A	*FADS2*	HDL2-C	6.58 × 10^−9^	0.43	Chr11-61,798,436-T-C	1.65 × 10^−8^	0.45	Yes, related trait	0.871
Chr11-105,327,888-G-C	*CARD18*	Triglycerides in medium VLDL	2.31 × 10^−9^	0.43	Chr11-105,331,384-A-T	1.05 × 10^−5^	0.43	No	NA
Chr18-49,653,146-G-A	*ACAA2, SMUG1P1*	Triglycerides in medium HDL	1.68 × 10^−9^	0.15	Chr18-49,642,278-G-A	4.71 × 10^−7^	0.16	Yes	0.864
Chr18-49,817,040-T-A	*SNHG22*	ApoA1	6.51 × 10^−9^	0.0041	Chr18-49,817,040-T-A^a^	2.15 × 10^−8^	0.0041	Yes	1
Chr19-44,922,203-A-G	*APOC1, APOC1P1*	Remnant-C	7.53 × 10^−11^	0.29	Chr19-44,922,203-A-G^a^	1.40 × 10^−9^	0.29	Yes	0.0783
Chr20-45,906,012-G-A	*PLTP*	Triglycerides in small HDL	1.80 × 10^−11^	0.23	Chr20-45,923,216-T-C	3.10 × 10^−10^	0.18	Yes	1
Chr21-16,318,536-C-T	*MIR99AHG*	HDL3-C	3.87 × 10^−9^	0.090	Chr21-16,318,536-C-T^a^	1.77 × 10^−8^	0.090	No	NA

Chromosome positions are based on GRCh38. For trait descriptors, see Supplemental Table S1, and for full results, see Supplemental Table S2. The genome-wide significance threshold is 7.17 × 10^−9^. To allow for a straightforward comparison, the LOCATER *P*-value was standardized to match the effective number of independent tests for single marker association.

^a^Denotes the associations that SMT lead marker is the same as LOCATER lead marker.

In terms of computational performance, we ran LOCATER using a total of 4780 jobs, each of which was assigned 12 cores and 60 GB of memory in an academic high-throughput computing system. On average, each job took ∼32 min. This shows the feasibility of running LOCATER at scale on real-world data sets. However, we note that LOCATER runtimes are sensitive to the number of genomic loci that show promising *P*-values during the initial screening procedure and will vary on a data set-by-data set basis.

**Figure 2.**
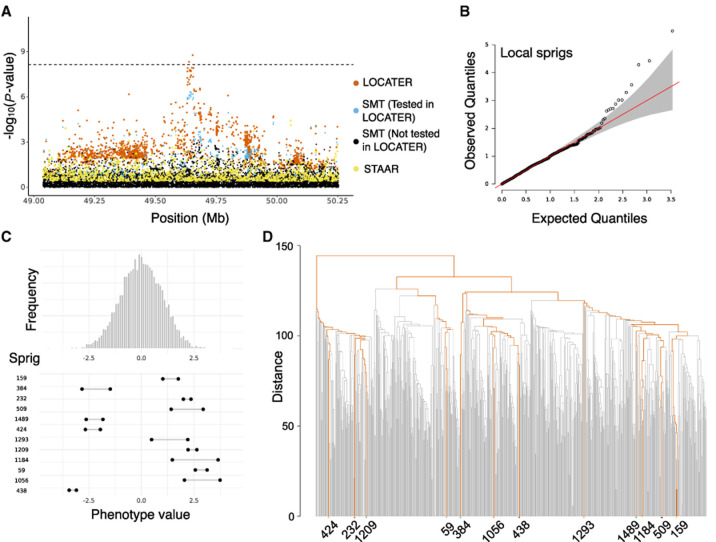
Association of triglycerides in medium HDL at *LIPG* locus. (*A*) Local Manhattan plot of the association signal for “triglycerides in medium HDL” on Chr 18: 49,038,347–50,253,146, including results for a single marker test (SMT; blue and black), LOCATER (orange), and STAAR (yellow). Note that LOCATER results are only shown for variants with an SMT *P*-value <1 × 10^−3^, because for computational efficiency only these variants were tested by LOCATER (see Methods). SMT results from variants tested by LOCATER are shown in blue, and those from variants not tested by LOCATER are shown in black. The black dashed line corresponds to the genome-wide significance threshold for SMT, standardized LOCATER, and standardized STAAR. (*B*) Q–Q inflation plot of −log_10_(*P*-values) from all “sprigs” at the lead marker Chr 18: 49,653,146, in which “sprigs” are defined as the smallest possible inferred clades. The gray area corresponds to the 95% confidence interval, and the red line denotes x = y. (*C*) Histogram of phenotype values after projecting out the genotype vector of the LOCATER lead marker (Chr 18: 49,653,146), thus removing signal that can be accounted for by the SMT subtest. Connected dots show the phenotype value of individuals assigned to significant sprigs. (*D*) Dendrogram generated from the haplotype-level local distance matrix at the lead marker Chr 18: 49,653,146. The UPGMA method was used for hierarchical clustering. Orange branches highlight the path of all haplotypes in significant sprigs shown previously in *C*. Labels at the *bottom* show the sprig assignment. For plotting clarity, 95% of haplotypes under insignificant sprigs were pruned.

Considering that this genome sequencing data set contains an abundance of rare variants, applying the canonical genome-wide significance threshold (5 × 10^−8^), which assumes 1 million independent tests, is not appropriate. We used permuted phenotypes to estimate the effective number of independent tests (see Methods) (Hoh et al. 2001; Gao et al. 2008). Based on the distribution of minimum *P*-values, we estimated that the effective number of independent tests for SMT (*T*_*SMT*_) is 6,977,438, and for LOCATER (*T*_*LOCATER*_) is 3,551,616 (α = 0.05). Based on *T*_*SMT*_, the genome-wide significance threshold for SMT should be 7.17 × 10^−9^. Note that LOCATER and SMT used the exact same set of variants, but the *P*-values reported by LOCATER are more dependent across loci owing to shared local genealogies. For a clearer comparison with SMT in visualizations (e.g., Fig. 2A), we further standardized the LOCATER *P*-value with

$$P_{LOCATER\;standardized}=P_{LOCATER\;modified\;GCed}\times T_{LOCATER}/T_{SMT}$$

to make the LOCATER genome-wide Bonferroni threshold match that of SMT (7.17 × 10^−9^). This does not change the interpretation of LOCATER results because both the results and the significance cutoff were rescaled to the same extent. To enable a direct comparison between SMT and LOCATER, all LOCATER results reported below are standardized.

After the screening, we identified loci of interest as genomic regions with one or more variants with significant *P*-values by either LOCATER or SMT in any of the 101 traits. To compare LOCATER and SMT signals at these loci, we defined a common shared association interval by merging the set of significantly associated variants identified by either method, in which the merging includes a 600 kb flanking region for each variant. For convenience, we used a similar process to merge association intervals across traits to obtain a nonredundant set of genomic regions, although in this case, we note that independent signals for different traits may often be lumped together (for the full set, see Supplemental Table S2). Altogether, we identified 47 genomic regions with 351 associations across all traits.

When comparing the most significant signal for LOCATER and SMT at every identified association (identified as min(*p*_*LOCATER standardized*_) < threshold or min(*p*_*SMT*_) < threshold), we found that LOCATER and SMT identified many associations together (327 out of 351) (Fig. 1D). Many of these are in canonical regions known to be associated with cardiovascular diseases, such as *PCSK9*, *APOB*, *LPL*, *LIPC*, *CETP*, and the *APOE/C1/C4/C2* gene cluster. A small number of associations are significant only in LOCATER (seven out of 351), and SMT found 17 associations that LOCATER did not (Fig. 1B).

Notably, in the cases in which SMT is more significant than LOCATER (321 out of 351 associations), it is typically by a very small margin, whereas in the cases in which LOCATER is more significant (30 out of 351), it is typically by a relatively substantial margin (Fig. 1C). Our interpretation of this result is that LOCATER has less power than SMT at trait associations resulting from a single causal variant because of the statistical penalty incurred by attempting to incorporate nearby signals but that LOCATER greatly outperforms SMT at loci with multiple causal variants. In total, five of the 47 significant loci (10.6%) show a signal boost from LOCATER, indicating that allelic heterogeneity is fairly common, even in a relatively small sample of the Finnish population that is known to be depleted of genetic diversity relative to most other human populations owing to historical bottlenecks.

For the 30 associations with a more significant signal from LOCATER, we inferred the number of independent causal variants based on the SD and QForm subtest signals from LOCATER (Supplemental Table S5). These 30 associations reside in 13 distinct genomic regions. After clumping associations that reside in the same genomic region (with 600 kb flanking regions) and involve traits that are highly correlated to each other (*r*^2^ > 0.8) in our data set, there were 21 nonredundant associations. Of these, the LOCATER signal boost came from SD in 15 cases and from QForm in six cases.

Of the 15 nonredundant associations that were boosted by SD, seven of them were boosted by only one sprig, whereas the other eight of them had two to 12 significant sprigs contributing to the signal. All significant sprigs represent distinct haplotype groups in the local ancestry trees. As a result, the total number of inferred causal variants for these 15 associations ranges from two to 13. We also report the variants that are completely linked with significant sprigs in Supplemental Table S4. For the six associations boosted by QForm, we used iterative conditional analysis to infer the number of causal variants. One association became insignificant after accounting for the lead marker, and signals in five other associations were diminished after multiple (two to five) rounds of conditional analysis, suggesting that the total number of inferred causal variants for these six associations ranges from one to five.

Rare variant association methods such as STAAR (Li et al. 2020) also seek to assess the combined effects of multiple causal variants at a locus, albeit using a very different approach than LOCATER. To test whether STAAR could potentially detect the same signals as LOCATER, we ran STAAR on the 30 associations mentioned above for which LOCATER had a more significant *P*-value than SMT. Notably, STAAR did not detect any of these 30 associations at genome-wide significance. A caveat to this analysis is that there are many different ways to run STAAR based on window size, variant inclusion, variant annotation, and weighting criteria, and so we cannot discount the possibility that STAAR might be able to detect some of these signals. For example, if STAAR is provided with variant impact scores from CADD (Rentzsch et al. 2019), it is able to identify four significant associations at the LIPC locus, albeit with far less significant *P*-values than SMT and LOCATER (more than 30 orders of magnitude difference). These results suggest that the trait association signals detected by LOCATER are not easily captured by current rare variant association methods such as STAAR. This is perhaps not surprising given that such methods are designed and primarily used for gene-based association, in which the target interval is well defined and variant annotations are much more informative.

Below, we discuss some of the trait associations detected by LOCATER. We highlight five known association signals that LOCATER detected but SMT did not, three cases in which both LOCATER and SMT detected the association but LOCATER provided a substantial boost in signal strength, and two potentially novel association signals detected solely by LOCATER.

### LOCATER recovers known associations at the *LIPG* locus

LOCATER recovered several known quantitative trait loci that would otherwise have been missed by SMT in our present study (see Fig. 1B), an example being the *LIPG* locus. *LIPG* encodes a well-known member of the triglyceride lipase family of proteins and is primarily involved in the metabolism of HDL (Jaye et al. 1999; Strauss et al. 2002; Jin et al. 2003; Ma et al. 2003). LOCATER recovered genome-wide significant associations for triglycerides in medium HDL (*P* = 1.68 × 10^−9^) and apolipoprotein A-I (*P* = 6.51 × 10^−9^), the major protein component of HDL particles. These two trait associations are likely to be independent given that the lead markers are in low LD (r^2^ of 7.29 × 10^−4^) and that the two traits are not significantly correlated (Pearson's correlation of 0.132), which is consistent with prior work (Davis et al. 2017). Neither of these associations was captured by SMT at genome-wide significance. The smallest SMT *P*-value for triglycerides in medium HDL within a 1.2 Mb window of *LIPG* was 4.71 × 10^−7^, and that for apolipoprotein A-I was 2.15 × 10^−8^ (Table 1). The lead variant for apolipoprotein A-I was also found in a prior study of Finns (*P* = 2 × 10^−10^) using many of the same METSIM samples analyzed here (Davis et al. 2017), and the lead variant for triglycerides in medium HDL was found in a large study of 233 metabolic traits in 33 cohorts (Karjalainen et al. 2024).

We first discuss the *LIPG* association with triglycerides in medium HDL, in which LOCATER detected a significant signal but SMT did not (Fig. 2A). LOCATER's improved power over SMT in this case comes from the SD subtest (Supplemental Fig. S5C,D), which indicates contributions from multiple ultrarare causal variants. We confirmed that the *P*-value distribution after modified GC aligns very well with the expected distribution, and the QQ-plot-based modified GC required to control confounding was minimal (Supplemental Fig. S5A,B).

The LOCATER SD subtest has the advantage that *P*-values from all predictors (sprigs, defined as the smallest possible inferred clades) are independent under the null hypothesis. A Q–Q plot of all −log_10_ sprig *P*-values calculated at the lead marker shows that the top 12 sprigs significantly deviated from the expected distribution and thus contributed to the SD signal (Fig. 2B). To highlight the coalescent path of significant haplotypes, we plotted a dendrogram of haplotypes based on hierarchical clustering of the similarity matrix at the lead marker, which showed that different significant sprigs were present within distinct clades in the local coalescent tree and that haplotypes in the same sprig were very similar. Haplotypes in the same sprig have a very recent coalescence, and haplotypes from different sprigs have a much more distant coalescence (Fig. 2D). This suggests that the SD subtest captured signals from multiple distinct haplotype groups rather than multiple signals driven by a single variant.

Notably, SD is able to combine signals from individuals at both extremes of the phenotype distribution, which correspond to alleles with opposing effects. As expected, all samples within significant sprigs have phenotype values that are far from the mean (Fig. 2C). Individuals within each sprig resided on the same side of the distribution, but different sprigs could reside on different sides.

We next sought to visualize variants influencing the SD signal at this locus using residual analysis. The first phase involved conducting SMT based on a phenotype that projected out the lead marker genotype vector, removing its effect on the signal. We defined the *P*-values resulting from this experiment as *P*_*S*_. The second phase involved performing SMT based on the residualized phenotype orthogonal against the lead marker SMT and SD signal, yielding a second set of *P*-values defined as *P*_*D*_. The difference between *P*_*S*_ and *P*_*D*_ shows the contribution of genomic variants to the SD signal. We plotted −log_10_ *P*_*S*_ and −log_10_ *P*_*D*_ on a Manhattan plot (Supplemental Fig. S5E) and a scatter plot (Supplemental Fig. S5F), highlighting variants in which *P*_*S*_ < 1 × 10^−3^ and *P*_*D*_ > 10 × *P*_*S*_. This experiment shows that SD captured signals from many variants scattered in an extensive genomic region (>10 Mb), supporting the success of our ancestry inference model tuning procedure.

By plotting PCs, we confirmed that SD-significant individuals do not form a tight cluster in any specific area of the plot, so this association signal was not obviously confounded by population structure (Supplemental Fig. S5G).

**Figure 3.**
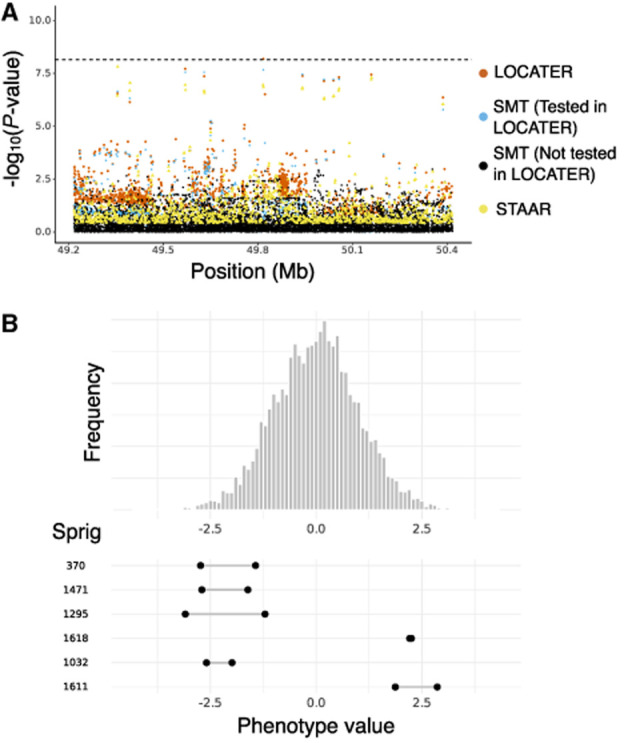
Association of apolipoprotein A1 at *LIPG* locus. (*A*) Local Manhattan plot of the association signal for apolipoprotein A1 on Chr 18: 49,217,040–50,417,040, shown using the exact same data types and color scheme as Figure 2A. (*B*) Histogram of phenotype values after projecting out the genotype vector of the LOCATER lead marker (Chr 18: 49,817,040), thus removing signal that can be accounted for by the SMT subtest. Connected dots show the phenotype value of individuals assigned to significant sprigs.

We now turn to the second *LIPG* association, apolipoprotein A-I. LIPG is known to regulate serum apolipoprotein A-I (Jaye et al. 1999; Ishida et al. 2003). A previous study in METSIM (Davis et al. 2017) showed that our lead marker is associated with five HDL subclass traits and apolipoprotein A-I (Fig. 3A), and in this study, LOCATER recovered the signal in apolipoprotein A-I, but SMT missed it.

LOCATER gained its advantage over SMT from SD (Supplemental Fig. S6A). The Q–Q inflation plot of sprig −log_10_ *P*-values showed that six sprigs contributed to the signal (Supplemental Fig. S6B). Similar to the first *LIPG* association result discussed above, haplotypes from the same sprig have a very recent coalescence; those from different sprigs have a more distant coalescence (Supplemental Fig. S6C); and samples within outlying sprigs had phenotypes that were far away from the median and on different sides of the distribution (Fig. 3B).

In summary, LOCATER was able to discover two independent trait associations at *LIPG* based on the presence of allelic heterogeneity, in which both trait associations were missed by SMT. In addition to these two examples at *LIPG*, three additional trait associations at other known loci were also detected by LOCATER but not by SMT. The association with HDL2 cholesterol on Chr 11 (Supplemental Fig. S13) and the association of monounsaturated fatty acids (MUFAs) on Chr 7 (Supplemental Fig. S9) were also identified based on SD signals and also showed consistent phenotype values across relevant individuals. Both associations were boosted by only one sprig, and phenotype values of individuals assigned to significant sprigs are all far from the mean of the corresponding phenotype distribution. There was also an association with “large VLDL particle concentration” on Chromosome 7 (Supplemental Fig. S11) that was boosted by QForm; however, this association is somewhat less confident than others given that only one variant crossed the significance threshold and that a substantial post hoc adjustment for confounding was required to calibrate the corresponding QQ-plot.

### A potentially new association on Chr 11

**Figure 4.**
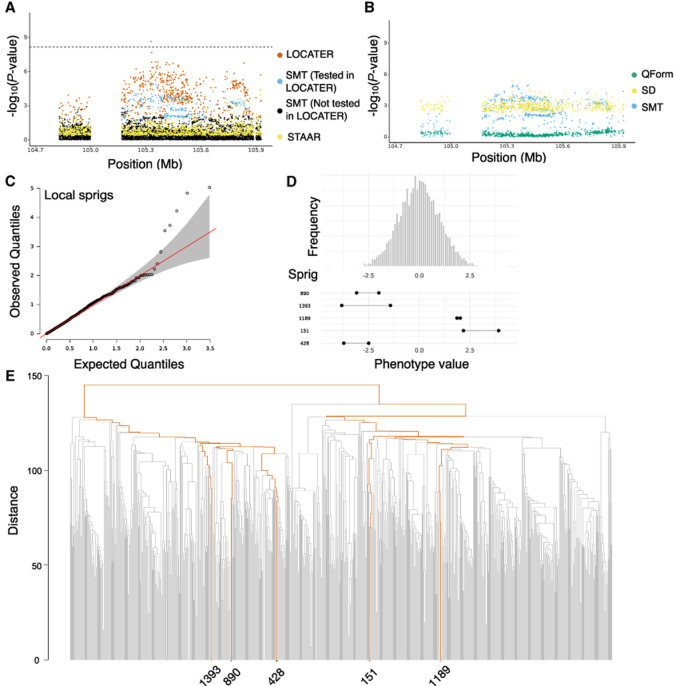
Association of triglycerides in medium VLDL on Chr 11. (*A*) Local Manhattan plot of the association signal for “triglycerides in medium VLDL” on Chr 11: 104,727,888–105,927,888, shown using the exact same data types and color scheme as Figure 2A. (*B*) Local Manhattan plot of “triglycerides in medium VLDL” at Chr 11: 104,727,888–105,927,888, showing modified genomic controlled −log_10_(P) for the three LOCATER subtests. (*C*) Q–Q inflation plot of −log_10_(*P*-values) from all “sprigs” at the lead marker Chr 11: 105,327,888, in which “sprigs” are defined as the smallest possible inferred clades. The gray area corresponds to the 95% confidence interval, and the red line denotes x = y. (*D*) Histogram of phenotype values after projecting out the genotype vector of the LOCATER lead marker (Chr 11: 105,327,888), thus removing signal that can be accounted for by the SMT subtest. Connected dots show the phenotype value of individuals assigned to significant sprigs. (*E*) Dendrogram generated from the haplotype-level local distance matrix at the lead marker Chr 11: 105,327,888. The UPGMA method was used for hierarchical clustering. Orange branches highlight the path of all haplotypes in significant sprigs shown previously in part *D*. Labels at the *right* show the sprig assignment. For plotting clarity, 95% of haplotypes under insignificant sprigs were pruned.

LOCATER found an association of “triglycerides in medium VLDL” on Chromosome 11, whereas SMT did not (Fig. 4A), and the GWAS catalog (Sollis et al. 2023) did not report any known association with correlated traits. LOCATER was much more significant than SMT owing to the SD subtest, implying a contribution from ultrarare haplotypes (Supplemental Fig. S7C). The SD signal was distributed evenly across the entire ∼1 Mb locus (Fig. 4B). Among all sprigs called at the lead marker position, the top five sprigs significantly deviated from the expected distribution (Fig. 4C). The phenotype distribution of individuals in significant sprigs showed that they are outliers and that trait outliers are found at both extremes of the distribution (Fig. 4D). The dendrogram from hierarchical clustering of the local similarity matrix, with highlighted coalescent paths for significant haplotypes, showed that the signal was not coming from a larger clade but rather from multiple distinct small groups of haplotypes (Fig. 4E). These results suggest that LOCATER is combining association signals from five rare haplotype groups with distinct genealogical histories, whereas these signals were not detectable by standard SMT.

We performed residual analyses to investigate the contribution of genomic variants to the SD signal and noticed many variants with drastically different *P*_*S*_ and *P*_*D*_ (i.e., substantial contribution to the SD signal). These variants extend across a megabase-long genomic distance, highlighting LOCATER's ability to merge subsignificant signals from a large genomic region.

We investigated this association and found that the lead marker resides in a predicted transcription factor (TF) binding site for KLF16 and KLF9. KLF9 is a metabolic TF acting in the liver, where its increased expression promotes gluconeogenesis and alters glucose and lipid homeostasis (Cui et al. 2019). The adjacent 1.2 Mb region includes CASP1, CASP4, CASP5, CASP12, CARD18, and CARD16. CASP1 (encoding caspase 1) has a core role as the central effector of the canonical inflammasome, and there is evidence from mouse studies that CASP1/inflammasome signaling can raise circulating TGs by increasing hepatic VLDL/APOB secretion and may also impair TG clearance via IL1-mediated suppression of LPL in adipose tissues (Bartolomé et al. 2008; van Diepen et al. 2013). The other genes in this region encode inflammasome-related caspases and CARD-family regulators, some of which enhance and others suppress CASP1-dependent inflammatory signaling. The genic content of this locus supports the plausibility of its association with “triglycerides in medium VLDL.” On the other hand, eQTLs for CASP1 and CASP4 have been identified in METSIM adipose tissue (Raulerson et al. 2019), but the lead eQTL variants are not strongly linked to the lead variant from LOCATER (*r*^2^ < 0.03) and are not associated with any traits in our study.

In addition, we detected a second potentially novel association with HDL3 cholesterol on Chr 21 (Supplemental Fig. S14). This association was attributable to SD combining signals from a single sprig. However, in this case, only one variant is significant, and even though the lead marker is genotyped in gnomAD, it resides in a 305 bp recent segmental duplication within which variant calling is likely to be prone to artifacts, making this result less confident than others presented here.

### LOCATER boosts a classic association at the apolipoprotein gene cluster

**Figure 5.**
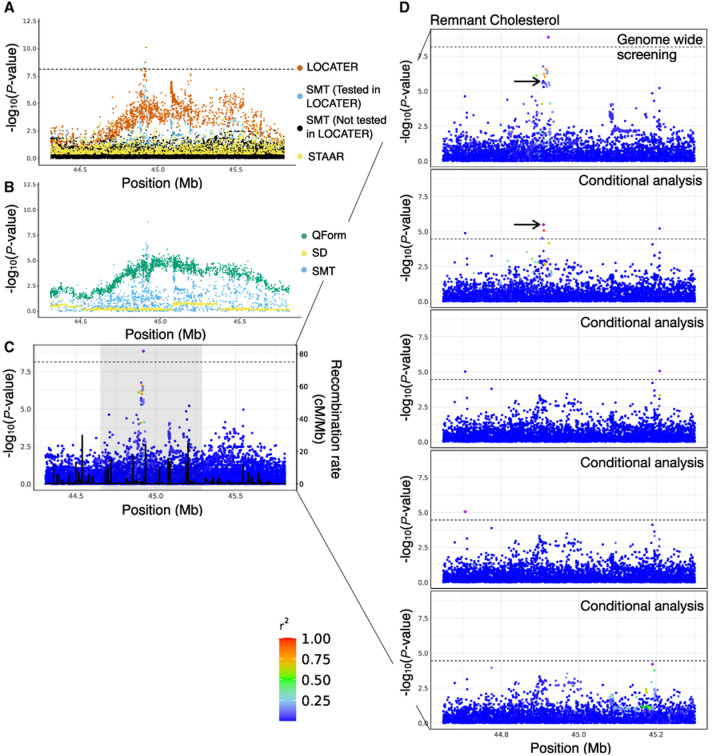
Association of remnant cholesterol at *APOE* cluster. (*A*) Local Manhattan plot of the association signal for remnant cholesterol on Chr 19: 44,308,684-45,809,149, shown using the exact same data types and color scheme as Figure 2A. (*B*) Local Manhattan plot showing modified genomic controlled −log_10_(P) for the three LOCATER subtests. (*C*) LocusZoom plot of SMT results. Variants are colored based on their *r*^2^ with the SMT lead marker Chr 19: 44,922,203 (purple diamond), in which LD is calculated in the studied samples. The black line shows the recombination rate in Finns (see Methods). Gene annotations are from GENCODE v45. (*D*) Zoomed in LocusZoom plots showing the original association at *top*, followed by the results from stepwise conditional analysis. Results were zoomed in based on the shaded region in *C*. Variants are colored based on their *r*^2^ with the SMT lead marker of each experiment (purple diamond). Black arrows point to the most significant variant from the GWAS catalog. The black dashed line corresponds to the genome-wide significance threshold (7.17 × 10^−9^) in the *top* panel, and the conditional analysis threshold (3.52 × 10^−5^) for the rest.

LOCATER recovered a known association for remnant cholesterol at the well-studied *APOE/C1/C4/C2* gene cluster on Chromosome 19 (Richardson et al. 2022; Karjalainen et al. 2024). This same locus is associated with various other traits that are correlated with remnant cholesterol (Supplemental Table S2). Although SMT also achieved genome-wide significance for remnant cholesterol, LOCATER's advantage over SMT implies that there are additional signals from other haplotypes (Fig. 5A).

In contrast to the other examples outlined above, LOCATER's advantage over SMT in this case came from QForm (Fig. 5B; Supplemental Fig. S8C), indicating that the causal haplotypes are likely to be more common (i.e., not ultrarare). Unlike the SD subtest, QForm does not inherently provide direct insight into the number of distinct haplotypes and how they relate to each other in the genealogy; however, using multiple rounds of conditional analysis with SMT, we confirmed that there are at least four groups of causal variants. We iteratively conditioned on the genotype vector of lead markers and observed that a significant association signal (*P* < 3.52 × 10^−5^) persisted through three rounds of conditional analyses (Fig. 5C,D). We found that the variants used as covariates in the conditional analyses are not in LD with each other, and one of them (Chr19-44908822-C-T) is the most significant known marker associated with remnant cholesterol (Fig. 5D, black arrow). These results show that LOCATER effectively combined signals from four distinct causal haplotypes, resulting in a substantial boost in power.

There are also two additional examples of known loci found by both LOCATER and SMT, in which LOCATER has a more significant *P*-value (implying the presence of multiple causal variants): the association with “triglycerides in small HDL” on Chromosome 20 (Supplemental Fig. S10) and the association of triglycerides in VLDL on Chromosome 7 (Supplemental Fig. S12). In both of these cases, the power boost was driven by the SD subtest.

## Discussion

We have used our new genealogy-based trait association method, LOCATER, to perform a genome-wide screen in a cohort of 6795 Finnish individuals with deep cardiometabolic trait measurements and WGS data. In total, we identified 30 associations at 13 known GWAS loci at which LOCATER was genome-wide significant and provided a clear power boost over SMT, seven associations of which (at five loci) were not genome-wide significant by SMT and would have been missed. LOCATER also identified two novel association signals, one of which is fairly compelling based on the underlying haplotype structure. At each locus, dissection of the association signals and underlying haplotype structure revealed evidence for allelic heterogeneity in the form of multiple independent association signals present in distinct portions of the local ancestry tree. Moreover, in the process of optimizing LOCATER's performance on real world genomic data, we made several key methodological improvements, including a novel approach for tuning ancestry inference parameters for trait association and a rigorous approach to account for the effects of cryptic confounders.

Genealogy-based trait association has been a topic of interest for nearly two decades. Seminal early work established its potential value using theory, simulations, and single-locus analysis (Zöllner and Pritchard 2005; Minichiello and Durbin 2006), yet the practical advantages of these methods are only now becoming accessible owing to recent advances in scalable tree inference (Kelleher et al. 2016; Speidel et al. 2019; Zhang et al. 2023; Gunnarsson et al. 2024; Wong et al. 2024; Deng et al. 2025), “clade association” methods capable of testing unobserved variants inferred by imputation (Zhang et al. 2023), and “global tree association” methods capable of combining association signals across multiple causal variants (Christ et al. 2022, 2026; Link et al. 2023; Zhu et al. 2024). In particular, our genome-wide analysis of 101 traits across 6795 individuals greatly exceeds the scale and statistical power of two prior efforts (Minichiello and Durbin 2006; Link et al. 2023) that applied global tree association to human data, both of which focused on smaller cohorts, a single trait, and a subset of the genome—a single locus in the former case and two chromosomes in the latter—and failed to detect any trait associations at genome-wide significance. More recently, Palamara and colleagues developed a new ARG inference engine and performed a gene-based association study with traits in the UK Biobank samples using a quadratic form test statistic (Gunnarsson et al. 2024; Zhu et al. 2024). This approach has impressive scalability and shows promise for improving gene-based testing; however, it remains unclear how this approach can be extended to genome-wide association testing while maintaining power and whether it is well calibrated on real-world traits in populations with complex or cryptic population structure. Here, we have used SD and QForm to perform global tree association genome-wide, at virtually all nonsingleton SNPs discovered by WGS; performed rigorous empirical correction for cryptic confounders; and identified hundreds of genome-wide significant trait associations. In the process, we encountered and overcame several key methodological obstacles related to parameter tuning and statistical calibration that have not previously been examined at this level of detail. These lessons are applicable to any haplotype association method, not just LOCATER, and thus, this work may serve as a roadmap for future efforts.

Our work also demonstrates that genealogy-based methods such as LOCATER show considerable promise for increasing the power of genetic association studies. LOCATER's ability to combine multiple signals resulted in more significant *P*-values over SMT at 8.5% of genome-wide significant associations and for 10.6% of loci, and the difference in *P*-value exceeded an order of magnitude difference in seven of these associations at five loci. The fact that such notable power gains are observed in the Finnish population, one of the least diverse human populations studied to date, suggests that our results are likely to be an underestimate of the performance improvements possible in more diverse populations that harbor more causal alleles per locus. LOCATER may be especially valuable for multiethnic studies, in which allelic heterogeneity is greatest and in which standard association methods have shown poor performance.

Relatively few of the 30 association signals for which LOCATER provided a power boost are likely to have been captured by other methods. None of these signals were captured by STAAR using a window-based screening approach that has been employed previously for rare variant association studies (although we note that STAAR is not explicitly designed or typically used for noncoding studies or for combining heterogeneous common variant signals). Moreover, although two recently developed methods (Link et al. 2023; Gunnarsson et al. 2024; Zhu et al. 2024) have some similarities with LOCATER, they rely on a quadratic form test for global tree association, whereas LOCATER uses SD in addition to QForm. We have previously shown via simulation-based studies that SD and QForm have complementary strengths and weaknesses: QForm excels when the causal variants are more common and reside further up in the tree, and SD excels at combining ultrarare variants residing in small clades at the bottom of the tree (Wang 2024; Christ et al. 2026). LOCATER tests SD and QForm sequentially, such that QForm is at a disadvantage for detecting signals. Nonetheless, given the complementary nature of the two tests, it is interesting to note that for the 21 nonredundant trait associations (accounting for correlated traits) in which LOCATER obtained more significant results than SMT alone, 15 were boosted by SD and six by QForm. This, along with our in-depth analysis of 10 loci, suggests that the SD test unique to LOCATER is providing substantial value beyond QForm by efficiently combining association signals across many clades. We expect the relative value of SD to increase as allelic heterogeneity increases, as in more diverse populations or for traits under negative selection. Despite these promising results, global tree association remains a difficult statistical problem, and we expect that future work in this area will continue to yield significant power improvements.

Tree-based trait association also presents unique challenges relative to SMT and gene-based testing. Perhaps the most difficult aspect of this study was the detailed work required to optimize haplotype inference and to control for cryptic confounders, both of which are important practical considerations for future studies. First, it is well known that the performance of the LS model HMMs is sensitive to the mutation and recombination penalty parameters, and prior studies have used different approaches for selecting them. We developed a novel tuning approach that optimizes regional trait association power at a distance of 0.05 cM rather than local variant imputation as in Relate (Speidel et al. 2019), and we explored a broad range of potential parameters to optimize haplotype inference for this specific population and data set. Optimizing for trait association power rather than imputation ensures that the resulting haplotype representations used carry long-range information required to combine independent signals at loci with allelic heterogeneity.

Second, we found that genealogy-based methods such as LOCATER are extremely sensitive to cryptic confounders, much more so than standard SMT, and that special measures are required to control for these effects. We studied this issue in detail and devised two new approaches to control for type I error: permuted rank matching of phenotype data to simulated Gaussian random variables to produce independent Gaussian phenotypes with minimal confounding, and a more general type of GC that fits both a slope and an intercept. In combination, these measures led to a well-calibrated analysis in our study and are likely to be applicable to future genealogy-based screens as well. The confounding effect of cryptic confounders was only apparent in our LOCATER analysis, whereas SMT appeared to be well calibrated after standard PCA-based measures. This result suggests that unadjusted confounders may plague SMT results to a larger extent than predicted from the body of QQ plots alone. We hypothesize that LOCATER is more sensitive to confounders than SMT because of the high correlation between proximal inferred genealogies along the genome. For intuition, consider the simple case in which there is a small subpopulation within a data set that has some environmental exposure that affects the phenotype of interest. There will be variants that tag that subpopulation throughout the genome. Every local ancestral tree inferred near those confounded variants will have a clade marking that subpopulation and thus have inflated test statistics. Thus, any potential confounders will affect LOCATER at many more markers than SMT, making the resulting inflation obvious in Q–Q plots.

Although there is more work to be done before these methods are mature, the work presented here, in combination with our prior simulation-based results (Christ et al. 2026) and recent work from others (Link et al. 2023; Gunnarsson et al. 2024; Zhu et al. 2024), suggests that genealogy-based trait association methods such as LOCATER are finally ready to fulfill their long-promised potential as practical tools for genome-wide association studies.

## Methods

### The METSIM study

METSIM is a single-site study investigating cardiometabolic disorders and related traits in 10,197 men randomly selected from the population register of Kuopio, Eastern Finland, aged 45 to 73 years at initial examination from 2005 to 2010 (Laakso et al. 2017). All participants provided informed consent. Following the exact procedure used by Locke et al. (2019), all raw phenotypes were adjusted for trait-specific background covariates. A rank-based inverse normal transformation was applied to the resulting residuals to obtain normalized phenotypes for association testing (for further details, see Locke et al. 2019). METSIM data are available from the database of Genotypes and Phenotypes (dbGaP: https://dbgap.ncbi.nlm.nih.gov/home/) under accession numbers phs001579 (genotype data) and phs000752 (phenotype data).

### WGS, data processing, and phasing

DNA samples were extracted from blood, and the DNA libraries were sequenced using Illumina HiSeq and NovaSeq systems, generating paired-end sequencing data. We performed sequence alignment and data processing with the “functional equivalence” pipeline (Regier et al. 2018) and performed variant calling with GATK (Van der Auwera et al. 2013), with modifications for computational efficiency. The variant call set QC steps were done rigorously to make sure only high-quality samples and variants were kept in the data set, and we performed phasing using Eagle2 (Loh et al. 2016) with strict parameters designed to ensure accuracy. The details of all the above steps are in the Supplemental Methods.

### Ancestral allele encoding

The Speidel version of the LS model (Speidel et al. 2019) requires ancestral allele information for ancestry inference. For this study, we used ancestral allele calls obtained via a 10-way EPO alignment of primates from Ensembl v106 (The 1000 Genomes Project Consortium et al. 2015). With BCFtools (Danecek et al. 2021), we updated the REF and ALT alleles and the genotype fields in VCF files to make the REF allele the ancestral allele. More details are in the Supplemental Methods.

### The LOCATER pipeline

Please see the “Code availability” section for a link to the code used in this paper. Briefly, the first step of LOCATER is to run local ancestry inference at each genetic marker included in the study. For this, we used the newest version (v2) of our local ancestry inference engine, kalis (Aslett and Christ 2024), which is an optimized implementation of the Speidel version of the LS model (Speidel et al. 2019). Notably, unlike other LS model implementations, kalis v2 uses an optimal checkpointing algorithm that allows it to be run on arbitrarily large sequences (e.g., whole chromosomes). Here, in the interests of computational efficiency, we only performed local ancestry inference on genomic segments that contained one or more single markers with a promising *P*-value (*P* < 10^−3^ in this study). In total, this included 5.7% of the genome. This step produces an N × N matrix (in which N is the number of haplotypes; 13,590 in this case) of genomic distances at each genetic marker.

The second step of LOCATER is to identify small clades, which we refer to as “sprigs.” LOCATER includes a sprig-calling algorithm that uses a multithreaded partial sorting algorithm to cluster the genomic distances into level sets separately for each haplotype, followed by a greedy clique–finding procedure to rapidly call sprigs. Then, at each genetic marker, LOCATER performs three types of association tests: (1) a standard SMT to measure the contribution of that specific marker, (2) SD to measure the contribution of the inferred small clades, and (3) a quadratic form–based test to measure the contribution of remaining ancestral relationships present at deeper portions of the tree (Christ et al. 2026). Steps 2 and 3 use a residualized phenotype vector from the prior step. This, combined with the independence guarantees of SD, ensures that the resulting three *P*-values at a given site are mutually independent under the null hypothesis. We then combine the three *P*-values using an adapted version of Fisher's method that we call Maximizing over Subsets of Summed Exponentials (MSSE) (Christ et al. 2026).

### Rank matching and selection

We ensured the Gaussianity of our phenotype vector by rank-matching our phenotypes to a vector of simulated independent Gaussian random variables. During preliminary analyses, we repeated this rank-matching process for the same phenotype several times, yielding a set of vectors, each a subtly different perturbation of the original phenotype. When we ran LOCATER across these different perturbations, the amount of inflation observed via the Q–Q plot of genome-wide LOCATER *P*-values differed moderately across perturbations (for examples, see Supplemental Figs. S2–S4). We believe this variation reflects the fact that, by chance, different perturbations can have stronger or weaker correlations with confounding processes such as population structure. Note that genome-wide SMT *P*-values are consistently well calibrated for all perturbations; thus, no adjustments were needed for SMT. Based on this observation, we simulated 100 perturbations of each phenotype and selected the perturbation that minimized the deviation from the expected tail distribution under the null hypothesis. Explicitly, for all perturbations of each phenotype, we ran LOCATER across evenly spaced variants (about 30,000 variants) along the genome, and after plotting the Q–Q inflation plot for −log_10_P of SD and QForm in LOCATER, we fitted a least-squares line over the *x* ∈ [2,2.5] domain of the resulting Q–Q inflation plot. This corresponds to fitting the tail of the null distribution based on all *P*-values in [0.01,0.0032]. We then used the parameters of each least-squares line (the slope and intercept) to select the perturbation that was closest to the expected null distribution. More explicitly, let the slopes of SD and QForm Q–Q plots be *m*_*SD*_ and *m*_*Q*_, whereas intercepts are *b*_*SD*_ and *b*_*Q*_, respectively. First, we selected all perturbations satisfying the following Boolean expression *m*_*SD*_ ∈ [0.8, 1.1] AND *b*_*SD*_ ∈ [–0.1, 0.1] AND ((*m*_*Q*_ ∈ [0.7, 1.2] AND *b*_*Q*_ ∈ [–0.1, 0.1]) OR (*m*_*Q*_ ∈ [0.6, 0.8] AND *b*_*Q*_ ∈ [0, 0.4])). If multiple perturbations met this requirement, the perturbation with the largest min(*m*_*SD*_, *m*_*Q*_) was chosen. If no perturbation met this requirement, which happened for 15 of our 101 phenotypes, the standard rank-based inverse normalized phenotype was used.

After the screening, we adjusted the *P*-value for each subtest based on the estimated tail parameters *m* and *b*, via

$$\begin{array}{rl} -\log(P_{SD\;modified\;GCed}) & =\frac{-\log(P_{SD})-\;b_{SD}}{m_{SD}}\;\mathrm{and}\\ -\log(P_{Q\;modified\;GCed}) & =\frac{-\log(P_{Q})-\;b_{Q}}{m_{Q}}. \end{array}$$

Because SMT is consistently well calibrated, no adjustments were applied to SMT *P*-values. To combine the resulting SMT, SD, and QForm *P*-values returned by LOCATER, we used the MSSE method in the work of Christ et al. (2026).

### Tuning ancestry inference for trait association

The LS model at the heart of the ancestry inference we used in this study, kalis, is a hidden Markov model (HMM) with two parameters that can be interpreted as tolerance for recombination and mutation, respectively. As we delineate below, rather than using EM or other more standard tuning objectives to select our recombination and mutation parameters, we chose parameters to optimize the propagation of proximal association signals along the genome in order to maximize LOCATER's power.

In our tuning procedure, we randomly sampled core genomic regions with at least 15,000 variants, each with flanking regions of 5000 variants on both sides. These flanking regions served as “burn-in” regions to ensure accurate ancestry inference along the full length of the core region. We then selected a variant in the middle of the core region as our target variant and used kalis to perform ancestry inference at that site.

We then selected a causal variant 0.05 (±0.005) cM away. In LOCATER, as in all GWAS studies, any association signals driven by variants that are colinear with the background covariates are assumed to be attributable to confounding processes. Thus, we only chose causal variants that were not colinear with the background covariates (multiple *r*^2^ ≤ 0.02). We then simulated a pseudophenotype vector with a strong effect driven by the causal variant. To ensure that the strength of this causal variant's signal (in terms of the observed −log_10_ *P*-value) would be roughly consistent across simulations, we increased the strength of the causal variant's effect as a function of the number of clades inferred at the target locus: More null clades yield a higher multiple testing burden for the causal variant to overcome.

Given our pseudophenotype vector, we ran LOCATER at our target variant *l*, yielding two *P*-values, $P_{SD}^{l}$ and $P_{Q}^{l}$. We assessed how these two *P*-values captured the signal at the nearby causal variant using

$$\frac{-\log(P_{SD}^{l}P_{Q}^{l})}{-\mathrm{lo}g(P_{SMT}^{m})}$$

as our relative signal metric. Here $P_{SMT}^{m}$ is the SMT *P*-value at the causal variant. We calculated this relative signal metric for 14 parameter settings (see Supplemental Table S3) across 144 distinct core regions sampled from our METSIM data set. Altogether, this procedure allowed us to select LS HMM parameters to maximize LOCATER's power in this METSIM study.

### Estimating the effective number of independent tests

Our data set has a huge number of rare variants; thus, the canonical *P*-value threshold of 5 × 10^−8^ is overly generous. Following the methods of prior work (Hoh et al. 2001; Gao et al. 2008), we estimated a genome-wide threshold to control the family-wise error rate at 0.05 by running our LOCATER method on 1000 simulated Gaussian phenotypes genome-wide. We ran SMT for each simulated trait and used the subset of variants with *p*_*SMT*_ < 10^−4.5^ for LOCATER association. The minimum *P*-value (*P*_min_) genome-wide was recorded for each simulated phenotype. Aggregating all 1000 simulations, we took the 95th percentile of the resulting *P*_min_ distribution as our genome-wide discovery threshold. Using this approach, we obtained 7.17 × 10^−9^ for *p*_*SMT*_ and 1.41 × 10^−8^ for *P*_*LOCATER*_ as the thresholds required to maintain a type I error rate of 0.05. To directly compare LOCATER and SMT, the final *P*-values of LOCATER were standardized so that its effective number of tests matches that of SMT, and both tests use the same threshold 7.17 × 10^−9^.

### Association screening

Because the Speidel version of the LS model requires ancestral allele calls (Speidel et al. 2019), we only included SNPs with ancestral allele calls in the 10-way EPO alignment of primates from Ensembl v106. Indels were ignored owing to their lower quality of phasing and variant calling. After removing monomorphic sites and singletons, we obtained a final data set of 18.9 million variants.

Kinship among individuals was calculated, and only unrelated individuals were included. For kinship analysis, we used the Hail function ‘hl.pc_relate’ (https://github.com/hail-is/hail) to compute pairwise kinship coefficients, and individual pairs with kinship ≥0.05 were flagged as related outliers. PCs were calculated in all Finnish samples, and a sample is considered a PC outlier and later removed if its score for any of the top 10 PCs falls outside the range of ±8 standard deviations from the mean score of that PC. This yielded a final data set of 101 traits and 6795 individuals. The top 10 PCs were included as background covariates in all association tests.

For computational efficiency, we adopted the following three strategies. First, we divided the whole genome into 4587 segments, with ± about 6000 variants of overlap between segments. The 4587 segment boundaries were defined so that each segment has a relatively similar number of variants to ensure similar run times during analysis. Second, although all of our variants were used for ancestry inference by kalis, we only ran LOCATER on a relatively small subset of target variants. We skipped variants in which the SMT *P*-values from all phenotypes were greater than 10^−3^ while ensuring that the recombination distance between consecutive target variants was at most 0.1 cM. Although selecting target variants in this way makes the resulting distribution of genome-wide *P*-values nonuniform, we do not use this distribution to estimate our GC parameters. Third, we avoided any expensive eigendecomposition steps in calculating *P*_*Q*_ at our target loci by using the Satterthwaite approximation to *P*_*Q*_ (Christ et al. 2026) in our first round of screening.

We identified any variant with a LOCATER combined *P*-value smaller than 7.17 × 10^−8^ in our first round of screening as putative variants. We then merged putative variants in all the phenotypes to generate putative loci (see main text) for follow-up. During association follow-up, we only focused on 10 cM regions centered around putative loci, which doubly ensured reliable ancestry inference. During LOCATER testing, we used partial eigendecomposition (computing only the matrix's largest magnitude eigenvalues and their corresponding eigenvectors) and χ^2^-based approximation (for details, see equation 11 in the methods of the work of Christ et al. 2026) of the remainder term implied by the local relatedness matrix to obtain precise *P*_*Q*_.

Because of the stochastic nature of the SD procedure, the *P*-values *P*_*SD*_ and *P*_*Q*_ returned by LOCATER are a function of the seed of the R environment. Setting the same seed for all segments along the genome is not recommended because it will cause additional correlation between variants, which will cause the *P*-value distribution to deviate from the expected uniform distribution. Instead, we strongly suggest setting different seeds for different segments along the genome. However, for reproducibility or for follow-up experiments of the same region or segment, we suggest using the same seed across experiments for consistent results. To assess the novelty of the association results, we used the GWAS catalog (April 22, 2024 version) (Sollis et al. 2023)

### Hierarchical clustering and visualization of local distance matrices

At loci with significant LOCATER associations driven by SD, we constructed a local tree based on the local relatedness matrix obtained by kalis to understand the relative placement of the sprigs driving that SD signal. Following the method used in the work of Speidel et al. (2019), we used mean-based hierarchical clustering (UPGMA) implemented in the ‘fastcluster’ R package (Müllner 2013). For plotting clarity, 95% of haplotypes under insignificant sprigs were pruned from the displayed dendrograms.

### Residual analysis to visualize SD signals

We employed a residual analysis procedure to visualize the association signals that contributed to the SD signal at a locus. Recall that LOCATER is a three-stage procedure in which a phenotype vector is passed between each step. To isolate the signals extracted by SD, we made a local Manhattan plot regressing the phenotype vector passed to SD (above we refer to the resulting *P*-value at a given variant as *P*_*S*_) and overlaid it with a Manhattan plot regressing the phenotype vector returned by SD (above we refer to the resulting *P*-value at a given variant as *P*_*D*_). We highlighted variants for which *P*_*S*_ < 1 × 10^−3^ and *P*_*D*_ > 10 × *P*_*S*_.

### Code availability

The code used to perform the analyses in this paper is available at GitHub (https://github.com/Xinxin-Wang-0128/LOCATER_real_data_vignette) and as Supplemental Code. The link to the LOCATER software is https://ryanchrist.github.io/locater/.

### Competing interest statement

The authors declare no competing interests.

## Supplemental Material

Supplement 1

Supplement 2

Supplement 3

Supplement 4
