# Supplementary material for: Genealogy-based trait association with LOCATER boosts power at loci with allelic heterogeneity
Source: Genome Res. 2026 May;36(5):976–91. doi: 10.1101/gr.280372.124 (PMC13445712; doi:10.1101/gr.280372.124)
Supplement: Supplementary file 2 [file Supplemental_Methods.pdf]

Supplemental Methods

Whole-genome sequencing, data processing, and variant calling

DNA samples were extracted from blood. We constructed DNA libraries with automated Kapa Hyper PCR free, automated TruSeq PCR free, Kapa Hyper PCR free, or TruSeq PCR free kit, with target insert size varying from 260 to 475. The libraries were sequenced with Illumina HiSeq 2000, 2500, X10, or NovaSeq 6000, generating 2 × 151-bp paired-end sequencing data.

We performed alignment and data processing using the "functional equivalence" pipeline (Regier et al. 2018). Briefly, we aligned reads to the GRCh38 human reference genome using BWA-MEM (v.0.7.15, Li 2013) and used Picard MarkDuplicates (v.2.4.1; http://broadinstitute.github.io/picard) to remove duplicate reads. We excluded samples that had estimated contamination >5% or that were likely to represent sample swaps (verifyBamID v.1.1.3, Zhang et al. 2020). We also required a discordant rate of <5%, haploid coverage ≥19.5X, inter-chromosomal rate of <5%, and first-of-pair mismatch rate of <5%.

We performed variant calling with HaplotypeCaller in GATK (v.3.5, Poplin et al. 2017) and concatenated the output into full single sample GVCFs (Picard MergeVcfs, v2.4.1, http://broadinstitute.github.io/picard). Since standard joint genotyping with GATK GenotypeGVCFs function could not scale to our CCDG callset, we used ReblockGVCFs (GATK v.4.2.2.0, Poplin et al. 2017) to decrease GVCF file sizes for future joint analysis in Hail. We used the ValidateVariants function to ensure the quality of the reblocking process. We then used the VariantDatasetCombiner function in Hail (v.0.2.78, Hail Team) to combine GVCFs from each sample into multi-sample VariantDataset (VDS) files before running GnarlyGenotyper

(unpublished version from Docker image gcr.io/broad-dsde-methods/gnarly_genotyper:hail_ukbb_300K, image hash ID: 7cc8cfa6e9af; created April 2020; received August 2021) for joint genotyping and VQSR to annotate variant quality. Finally, we converted VDS files into MatrixTable (MT) files (Hail v.0.2.97, Hail Team) and decomposed multi-allelic variants into bi-allelic variants.

To QC the variant callset, we excluded samples that had a low het/hom ratio (<5 MADs less than the median), low sequencing depth (number of bases with depth >10 is <20 MADs less than the median), or excessive number of singleton variants (>20 MADs more than the median), where each of these criteria was applied separately to each self-reported ancestry group. We also removed samples with fewer than 580,000 insertions or deletions in joint variant calling, genetic-phenotypic sex mismatches, withdrawal of consent, sample swaps, or inheritance inconsistencies and other sample identity issues. The maximal independent set of these samples was calculated in Hail (Hail Team) using `hl.maximal_independent_set` and individuals up to second-degree related were removed. For variant level QC, we flagged genotype calls with genotype quality (GQ) < 20, depth (DP) < 10, and heterozygous calls with allele balance (AB) ≤ 0.2 or ≥ 0.8 as low quality, and filtered out variants that had AS_VQSLOD < 0 or that had a high proportion (>95%) of missing or low quality genotypes.

Phasing

We performed a more stringent variant quality control for phasing. After exporting Hail MT files to VCFs (Hail 0.2.95), we selected PASS and non-singleton variants and filtered out sites with high quality genotype call rate < 90% or Hardy-Weinberg $p < 10^{-7}$ (one-sided p-value for excess

heterozygotes). METSIM samples were phased with other WashU CCDG samples without a reference panel with Eagle2 (v.2.4.1, Loh et al. 2016). Due to memory restraints, we divided chromosomes into 20 Mb chunks with 2 Mb overlaps on both ends. Phasing was done with default options except for a bigger `Kpbwt` value (200k) for better phasing accuracy. Missing genotypes were imputed during phasing.

Rare variant association experiment with STAAR

We applied STAAR (Li et al. 2020) with the sliding window method using default parameters. The window size is 2 kb and the sliding step length is 1 kb. For each window, we included all rare variants with AF < 0.01 and removed any window with fewer than two rare variants. We reported the STAAR-O p-value.

Ancestral allele encoding

We downloaded the human ancestral genome FASTA from 10-way EPO alignment of primates from Ensembl v106 available at https://ftp.ensembl.org/pub/release-106/fasta/ancestral_alleles/homo_sapiens_ancestor_GRCh38.tar.gz created on March 19, 2022. In the FASTA file, lowercase indicates lower quality. For simplicity, all lowercase letters were converted to uppercase. Since the conversion works best when multiallelic variants are merged, we merged them with `bcftools norm -m +any` (BCFtools v1.9, Danecek et al. 2021). We then used `bcftools norm --check-ref s --fasta-ref {fasta_file} ` (BCFtools v1.9, Danecek et al. 2021) to edit the REF allele in VCF files to be the ancestral allele, which automatically updated the genotype field. The files also went through `bcftools +fill-tags` (BCFtools v1.16, Danecek et al. 2021) to make sure the AC tag of the INFO column in VCF files

is correct. Finally, we split multiallelic sites into biallelic variants with `bcftools norm -m -any` (BCFtools v1.16, Danecek et al. 2021).

REFERENCES

Danecek P, Bonfield JK, Liddle J, Marshall J, Ohan V, Pollard MO, Whitwham A, Keane T, McCarthy SA, Davies RM, et al. 2021. Twelve years of SAMtools and BCFtools. *Gigascience* **10**: giab008.

Hail Team. Hail 0.2. https://github.com/hail-is/hail.

Li H. 2013. Aligning sequence reads, clone sequences and assembly contigs with BWA-MEM. *arXiv [q-bioGN]*. http://arxiv.org/abs/1303.3997 (Accessed February 1, 2026).

Li X, Li Z, Zhou H, Gaynor SM, Liu Y, Chen H, Sun R, Dey R, Arnett DK, Aslibekyan S, et al. 2020. Dynamic incorporation of multiple in silico functional annotations empowers rare variant association analysis of large whole-genome sequencing studies at scale. *Nat Genet* **52**: 969–983.

Loh P-R, Danecek P, Palamara PF, Fuchsberger C, A Reshef Y, K Finucane H, Schoenherr S, Forer L, McCarthy S, Abecasis GR, et al. 2016. Reference-based phasing using the Haplotype Reference Consortium panel. *Nat Genet* **48**: 1443–1448.

Poplin R, Ruano-Rubio V, DePristo MA, Fennell TJ, Carneiro MO, Van der Auwera GA, Kling DE, Gauthier LD, Levy-Moonshine A, Roazen D, et al. 2017. Scaling accurate genetic variant discovery to tens of thousands of samples. *bioRxiv* 201178. https://www.biorxiv.org/content/10.1101/201178v3.abstract (Accessed February 1, 2026).

Regier AA, Farjoun Y, Larson DE, Krasheninina O, Kang HM, Howrigan DP, Chen B-J, Kher M, Banks E, Ames DC, et al. 2018. Functional equivalence of genome sequencing analysis pipelines enables harmonized variant calling across human genetics projects. *Nat Commun* **9**: 4038.

Zhang F, Flickinger M, Taliun SAG, InPSYght Psychiatric Genetics Consortium, Abecasis GR, Scott LJ, McCaroll SA, Pato CN, Boehnke M, Kang HM. 2020. Ancestry-agnostic estimation of DNA sample contamination from sequence reads. *Genome Res* **30**: 185–194.
